# Worlds Apart, Te Ao Māori and Western Worldviews in Aotearoa, New Zealand

**DOI:** 10.1007/s11673-025-10439-2

**Published:** 2025-06-20

**Authors:** A. Hikairo Spelman, B. V. Dieriks

**Affiliations:** 1 Faculty of Science, Psychology, University of Auckland, Private Bag 92019, Auckland, 1142 New Zealand; 2 Department of Anatomy and Medical Imaging, the University of Auckland Private Bag 92019, Auckland, 1142 New Zealand; 3 Te Huinga Hinengaro—Centre for Brain Research, University of Auckland, Private Bag 92019, Auckland, 1142 New Zealand

**Keywords:** Worldviews, Te Ao Māori worldview, Western worldviews, Māori, Te Tiriti o Waitangi

## Abstract

Even though the last war between Tangata Whenua and the Crown ended over 150 years ago, Tiriti obligations and the rights of Tangata Whenua remain largely unaddressed. Significant disparities persist, with limited discourse on effective solutions. The recently introduced Principles of the Treaty of Waitangi Bill highlights enduring challenges in honouring Te Tiriti o Waitangi. This manuscript examines Te Ao Māori (the Māori worldview) and Western worldviews in Aotearoa, exploring their profound cultural differences and implications for relationships between Tangata Whenua and Tangata Tiriti. We highlight the holistic, interconnected nature of Te Ao Māori, rooted in whakapapa, and contrast this with the rationalism and compartmentalization of Western traditions. To bridge these worldview differences, we propose a relationship framework grounded in Te Tiriti o Waitangi to promote equitable, respectful partnerships. This framework addresses power imbalances and advocates for a two-worldview methodology that honours the mana of both perspectives. By integrating these approaches, we identify pathways for building stronger, inclusive relationships. This pluriversal approach respects the integrity of both worldviews and offers a foundation for coexistence rooted in mutual respect.

## Introduction

In this manuscript, we explore the origins and development of worldviews and their importance in shaping the way we express ourselves culturally through values and beliefs, which are found to be operating at all levels of society. We can access a Tangata Whenua worldview by exploring Te Ao Māori and a Crown worldview by accessing that broad set of values and beliefs that fall under the heading of “Western Worldview.” Our colonization history in Aotearoa is a story of Western Worldview domination, and if we are to correct this situation, we must first be clear about the key components of each worldview.

Te Ao Māori is characterized by interconnectedness and collaborative practice that transcends tribal boundaries (Mikaere 2011; Edwards et al. 2023; Royal 2003). In contrast, a Western Worldview operates by segmenting the whole as we experience it in order to make sense of the parts. The aim is to examine how individuals or groups from different worldview perspectives can form cooperative relationships without prioritizing one worldview over the other. This is a challenge that remains highly topical and relevant in present-day Aotearoa (Los 2023). Next, we discuss some critical issues involved in working with worldview differences and in the final sections, we review approaches to managing worldview differences in Aotearoa, proposing methods to improve interactions between Tangata Whenua and Tangata Tiriti in groups and organizations within the Tangata Whenua, Community and Voluntary Sector, and the Public Sector in Aotearoa.

Te Tiriti o Waitangi positions us for the challenge of dealing with worldview differences as we communicate across the gap between the two parties. As a Māori worldview differs greatly from a Western worldview, a specific methodology is needed to facilitate connection and communication. Acceptance of worldview difference rather than sameness is crucial for operating a methodology with credibility in a Tiriti two-worldview setting. When one party dominates or uses elements of their worldview to force change on the other, there is a loss of mana on both sides. Working together using a Tiriti two-worldview approach keeps the mana of both worldviews intact and on par with each other. It discourages a “winners and losers” mentality between the worldviews where tension arises. It also discourages assimilation (Patterson 1992; Barlow 2002; Royal 2003; Hikuroa 2017; Mikaere 2011; Edwards et al. 2023).

We explore how worldview differences are reflected in identity, examining the role of understanding “who we are” in shaping values and assumptions within relationships. Relationship breakdowns are often rooted in an inability to handle or navigate these differences (Shiundu 2024). Drawing on both Māori and Western traditions, we trace the historical and philosophical roots of these perspectives. Kamler and Thomson’s (2006) view of doctoral research as a social practice aligns with this relational focus, emphasizing the integration of identity, process, and outcomes in fostering meaningful engagement. Such relational methods support the Tiriti-based approach necessary for building effective, respectful partnerships.This approach is particularly relevant to groups where fostering mutual understanding and collaboration is crucial. By integrating relational approaches that respect and uphold the integrity of both worldviews, this manuscript contributes to the broader dialogue on creating equitable, inclusive, and cooperative relationships across our public life in Aotearoa (Edwards et al. 2023; Walker, Reedy, and Tibble 2022).

## Te Ao Māori—Māori Worldview

Te Ao Māori is grounded in an interconnected framework of relationships that provide meaning and structure to Māori thought. This worldview explores cosmogony (origins of the universe), ontology (the nature of existence), epistemology (knowledge acquisition), teleology (life’s purpose), and ethics, integrating spiritual, physical, and moral dimensions (Royal 2003; Barlow 2002; Mikaere 2011; Patterson 1992). Unlike Western empirical approaches, which often isolate knowledge into discrete parts, Te Ao Māori emphasizes the nature of understanding, where everything is approached relationally and where knowledge is as much subjective as it is objective. Royal (2005) describes knowledge from a Māori perspective as the internal consciousness of a person. He contrasts this with its Western equivalent, where knowledge becomes the product of consciousness. Royal adds that experience in Te Ao Māori is inseparable from knowledge whereas within a Western paradigm, knowledge becomes the explanation of experience (Royal 2005). Central to Te Ao Māori is *whakapapa* (genealogy), which connects all living and non-living things through layered relationships. Whakapapa provides a framework for understanding the universe’s interconnected realms: *Te Kore* (potential), *Te Pō* (becoming), and *Te Ao Mārama* (being). These genealogies, embedded in creation stories, guide ethical behaviour and reflect the intrinsic link between human well-being and environmental sustainability (Reilly et al. 2018; Royal 1998).

## Key Kaupapa and Tikanga from Te Ao Māori

The following elements of Te Ao Māori have been chosen because of their relevance to working with groups, organizations, and communities. They are discussed in the context of an interrelated whole.

*Manaakitanga* (hospitality) is central to fostering and maintaining relationships, where care and respect for others enhance the reciprocal nature of mana (prestige and authority). This value is vital in whānau (family) and marae (meeting space in front of the wharenui), underscoring importance of mana-enhancing relationships as a cornerstone of social cohesion (Kawharu, Tapsell, and Tane 2024). *Whanaungatanga* (kinship) builds upon whakapapa, focusing on the bonds and mutual obligations within whānau, hapū (sub-tribes), and iwi (tribes). It also reflects the collective nature of Māori society, where the value of relationships extends beyond human connections to include the environment and spiritual entities, reinforcing the concept of interdependence. The deep relationship between people and the environment is expressed through *mana whenua*, which links spiritual authority to land. Practices like burying the placenta in the whenua symbolize this connection and the reciprocal responsibilities between people and the natural world (Read 2021).

Māori values are further shaped by *atuatanga* (divine excellence), representing life’s ultimate purpose. This spiritual dimension is aligned with a holistic set of interconnections between the spiritual, social, and material dimensions, promoting harmony across all aspects of existence (Royal 2003). *Tikanga Māori* (customs) provides practical and ethical guidance for daily life. While it evolves over time, it retains core practices arising from the distinction between tapu (sacred) and noa (ordinary), which continue to influence how respect for food-related spaces is valued (Rolleston, McDonald, and Miskelly 2022). Tikanga fosters sustainable practices, ensuring that actions align with cultural values and obligations to people and the environment. *Kaitiakitanga* (guardianship) exemplifies the Māori stewardship ethic derived from whakapapa and the interconnected relationships among all living things. Kaitiaki (mana whenua guardians) actively maintains the balance within the natural world, ensuring that resources and ecosystems are protected for future generations (Roberts et al. 1995; Resource Management Act 1991). *Kotahitanga* (unity) emphasizes the importance of collective action and harmony within communities. By discouraging conflict and division, kotahitanga guides cohesive relationships that align with the understanding of the Māori world and its interconnected parts (Bishop 2008). Finally, *rangatiratanga* (sovereignty) embodies leadership and self-determination grounded in relational ethics. It reflects authority within the framework of Te Tiriti o Waitangi and provides a governance model that respects the unity and diversity of communities in Aotearoa (Hēnare 2015).

The Māori worldview, anchored by values such as manaakitanga, whanaungatanga, and kaitiakitanga, illustrates the intrinsic interdependence of people, the environment, and spiritual dimensions. These values collectively reinforce the nature of Te Ao Māori, where relationships define identity and guide ethical action. In a modern context, this perspective offers profound insights for fostering sustainable, inclusive practices that address contemporary societal challenges in Aotearoa and beyond.

Kaupapa Māori frameworks have been effectively implemented across various organizations and community initiatives, including the Community Sector Taskforce, Counties Manukau District Health Board, Housing New Zealand Corporation, the Māori Business Network, Te Wānanga o Raukawa, and the Māori Party. Models such as *Te Whare Tapa* *tWhā*, *Te Wheke*, and *Ngā Pou Mana* exemplify interconnection. *Te Whare Tapa Whā* conceptualizes health through the metaphor of a house with four dimensions—physical, mental, family, and spiritual—emphasizing the need for balance across these aspects (Durie 2004). *Te Wheke*, represented by the octopus, also highlights interconnectedness, with the tentacles symbolizing dimensions such as life force (mauri), ancestral connection (hā a koro mā a kui mā), and emotional expression (whatumanawa), suggesting that imbalance in any dimension impacts overall well-being (Pere 1984). *Ngā Pou Mana*, introduced by the Royal Commission on Social Policy, uses four foundational pillars—family (whanaungatanga), cultural heritage (taonga tuku iho), the environment (te ao tūroa), and land base (tūrangawaewae)—to provide a comprehensive framework for community and individual health planning (Durie 2004). The Whānau Ora initiative further exemplifies kaupapa Māori by empowering families to achieve better health and social outcomes. It connects applications of Māori values to the way service delivery can be changed, focusing on the interdependence of physical, mental, spiritual, and social health to support holistic family well-being.

In summary, Te Ao Māori, as a worldview, presents itself as an all-encompassing philosophical framework. It facilitates inquiries into identity, origins, the nature of the world, our place in the universe, our future direction, knowledge acquisition, the development of ethical conduct for enduring physical and cultural existence, and innovative responses to emerging challenges (Mikaere 2011; Royal 2002).

## Tangata Tiriti Worldview

Understanding the Tangata Tiriti worldview is crucial for engaging meaningfully with the differences between Tangata Whenua and Tangata Tiriti perspectives in Aotearoa, New Zealand. Grounded in Western traditions, this worldview emphasizes historical and conceptual approaches that often contrast sharply with Māori perspectives, particularly regarding land and nature. While Māori view land as sacred, embodying stewardship (*tiakitanga*), the Western tradition has often regarded it as a commodity, reflecting a broader disconnection between humanity and the natural world (Williams, Roberts, and McIntosh 2012; Berry 1988; Webster and Cheyne 2017).

Western Worldview thinking has evolved over the centuries. Karl Jaspers wrote about an Axial Period (800–200 BCE) which marked a significant shift in global thought, giving rise to new philosophical traditions and major ideologies, such as Greek rationalism and monotheism. This period deepened the separation of humanity from nature and individuals from one another, profoundly influencing Western cosmology and societal organization (Armstrong 2006; 1999; Jaspers 1953). Over time, Western thought evolved through three key epochs: Greek rationalism in the classical era, Christian theology in the medieval period, and empirical science in the modern era. Each phase built upon the intellectual legacy of the Axial Period, progressively shifting toward a fragmented, human-centric view of the universe. By the modern era, Cartesian dualism reinforced a division between mind and matter, prioritizing scientific inquiry and individual rationality while diminishing the importance of maintaining links between spiritual and natural dimensions of the wider world (Tarnas 2010). Romanticism in the late eighteenth century countered this trend by celebrating emotional depth, artistic creativity, and humanity’s connection to nature. David Bohm’s theory of the “implicate order” further challenged the dominant fragmentation of the Western Worldview proposing a holistic view of reality all elements of the universe are connected (Bohm 1980). This holistic perspective offers an alternative to the mechanistic and reductionist approach prevalent in Western thought.

In a contemporary Western worldview, tensions persist between fragmentation and integration. Postmodernism emphasizes the fluidity of knowledge and experience, advocating for diverse approaches to truth and rejecting fixed principles. Participatory epistemology seeks to reconcile this divide by presenting the human mind as a tool for articulating interconnected realities, fostering a holistic and participatory worldview (Berry 1988). The dominance of a fragmented, mechanistic understanding in Western approaches often conflicts with nature-centric and relational perspectives. Concepts such as Freud and Jung’s focus on the unconscious, along with participatory methodologies in modern research, highlight the potential for reconnecting individuals with the cosmos and adopting a more nature-centric approach (Tarnas 2006). Ultimately, while the Western tradition has profoundly shaped Tangata Tiriti perspectives, the need to connect relational and ecological approaches is increasingly recognized. This shift aligns with the broader quest to reconcile fragmented Western worldviews with holistic frameworks better suited to addressing contemporary challenges.

## Discussion

Western methodological thought, traditionally anchored in individualism from the perspective of Descartes’ “I think therefore I am,” contrasts sharply with the collectivist values of other cultural paradigms (Friston 2016; Gergen and Gergen 2003). The discourse around the African philosophy of Ubuntu, translated as “I am because we are,” asserts that the individual’s existence and knowledge are contingent upon relationships with others (Battle 2009). This aligns with the concept of “double consciousness,” as described by W.E.B. Du Bois, which refers to the experience of black Americans feeling a sense of “two-ness” in their soul, thoughts, and perceptions. This term articulates the internal conflict of navigating a bicultural framework relating to identity, primarily from an individual perspective (Ladson-Billings 2003).

In Aotearoa, the interaction between a Western worldview and Te Ao Māori within a decolonization framework calls for a pluriversal approach that acknowledges the legitimacy and richness of diverse epistemic, ethical, and political thinking and practice from both worldview perspectives. Grosfuguel’s advocacy for a pluriversal world instead of a singular universal design reflects a critical dialogue essential for honouring both Tangata Whenua and Tangata Tiriti perspectives (Jaramillo and McLaren 2008). This indigenous challenge to universalism, powerfully rooted in the colonization discourse, critiques the Western worldview for its historical tendency to segment knowledge and relegate non-Western epistemologies to “myth,” a category deemed irrational by thinkers like Habermas (Mutu (Ngāti Kahu) 2019).

Acknowledging Te Ao Māori and challenging Western universalism underscores the importance of engaging with the complexities of knowledge creation and understanding from both worldview perspectives, not just one. The shift towards a more inclusive and relational approach in both research and public life emphasizes not only the interconnectedness of body, mind, and spirit but also the multiple interconnections across the wider natural world. Such an approach offers a holistic perspective that transcends the transactional segmentation of Western thought, promoting a comprehensive view that includes Te Ao Māori in a way that avoids “problematizing the indigenous” or assimilation (Aluli-Meyer 2008; Wilber 1983; 1995; Smith 1999). As Aotearoa moves towards a post-colonial environment, effective engagement between Tangata Tiriti and Tangata Whenua worldviews will be needed. Successful engagement will require support from a broad and well-defined analytical framework based on Te Tiriti o Waitangi that respects the rich diversity and historical experiences of people in both communities.

Te Tiriti o Waitangi Relationships Framework encourages dialogue across significant worldview differences, fostering a meaningful basis for developing change that respects worldview differences that arise when the Western tradition and Te Ao Māori engage each other. Such an approach is necessarily collaborative and essential for tackling contemporary challenges across communities to ensure the sustainability of agreed change action.

**Figure 1:**
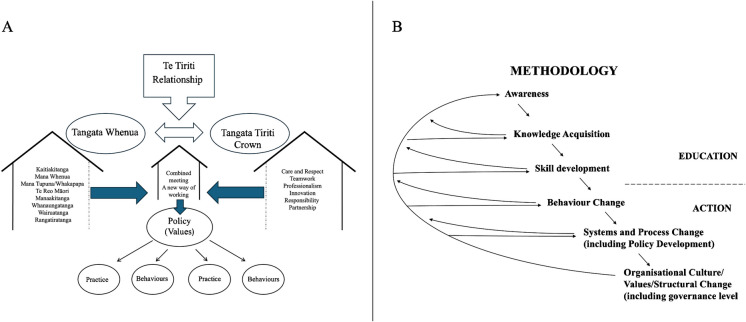
**(A)** Two-worldview behavioural application Tangāta Whenua and Tangāta Tiriti Values relationship framework and **(B)** Methodology of development and change

In Aotearoa, over the last forty years, our track record for dealing with worldview differences when deciding how we will manage community governance has been shaped by the colonization agenda and academic critique/challenge from a Māori perspective where Te Ao Māori is not operating on its own terms. This is likely because the methods of analysis, decision-making, and implementation of change require change. The work of the Waitangi Tribunal (Came, Sullivan, and Kidd 2020), notwithstanding that it is a Crown agency marked the beginning of an iterative change process that has enabled us to progressively develop our understanding of a different type of methodology (Figure 1).

The work of the Waitangi Tribunal has, from 1975, raised the level of awareness of Tangata Tiriti to the possibility that there might be another way of looking at the world rather than through a Western lens. The effect of expanding, in 1985, the scope of the Tribunal’s enquiries back to 1840 has expanded our collective understanding of the history of Aotearoa, and particularly our cultural history. That this process was iterative does not lessen its value as an approach to dealing with this type of change. A careful reading of the many reports of the Waitangi Tribunal illustrates the progressive workings of a different methodology that, we believe, has the potential to deliver better change actions than a methodology driven predominantly by a Crown worldview.

Understanding the journey since 1840 differs from comprehending the direction forward. The tensions between Articles 1 and 2 of Te Tiriti o Waitangi highlight two key points: first, the different worldviews of Tangata Whenua and the Crown are in the 1840 document; second, these tensions are not to be “resolved” or the Articles rewritten. Recent developments, such as the introduction of the Treaty Principles Bill by ACT Party leader David Seymour, have intensified these discussions. The bill aims to redefine the Treaty’s principles, sparking significant debate and public protests, including a notable haka led by Māori MPs in Parliament (Seymour 2024). These events underscore the relevance and complexity of interpreting Te Tiriti in contemporary Aotearoa.

To continue to honour Te Tiriti as the nation’s founding document, we must develop robust mechanisms that respect and advance the interests of both parties, embracing the inherent tensions as integral to the partnership it establishes. A relational framework based on Te Tiriti o Waitangi can help us undertake change that is relevant, respectful, and sustainable. We propose a relationship framework that focuses on driving change but acknowledges the need for prior learning. A Tiriti/Treaty two-worldview approach builds on understanding worldview differences and facilitates analysis beyond mere critique. It begins by engaging both worldviews to foster mutual understanding. Learning and development processes must be tailored to the specific group involved, with adjustments in design and direction that suit the context (Figure 1A) (Smith 1999).

The proposed methodology guides movement across six continuum points, balancing learning with action (Figure 1B). Each step assumes competence at prior levels (Freire 1996). Both parties introduce their worldviews, and after thoroughly discussing how each value under consideration is applied to the issue at hand, they can attempt to look at the question of change because both sides will have a shared understanding of what they are dealing with. The shared application of values to the issue at hand will be crafted in such a way as to ensure the integrity of each worldview, especially Te Ao Māori (Royal 2002). This is broadly the process of engaging a Tiriti/Treaty two-worldview in the workplace or the community with the support of an appropriate methodology.

This practical approach bridges cultural theory and application, embracing dialogue between Tangata Whenua and Tangata Tiriti perspectives. It focuses on the behavioural application of values to facilitate the creation of a shared commitment to making changes that strengthen the Tiriti relationship, not weaken it.

## Conclusions

The journey towards a genuinely inclusive and pluralistic Aotearoa necessitates a more profound engagement between Tangata Tiriti and Tangata Whenua perspectives than we have experienced. This engagement must transcend historical paradigms and embrace a future where diverse worldviews coexist and enrich each other, forming a unique tapestry of knowledge and understanding. The dialogue must be rooted in mutual respect, acknowledging the sovereignty and integrity of Te Ao Māori while appreciating the multifaceted nature of Tangata Tiriti worldviews.

Critical to embracing a pluriversal approach is the need to champion the co-creation of knowledge through critical dialogue across diverse epistemic, ethical, and political landscapes. This approach recognizes the limitations of a singular, universal perspective and opens the door to a more nuanced, collective pursuit of understanding.

Institutional and societal structures within Aotearoa must reflect this commitment to inclusivity, ensuring that policies, practices, and educational curricula are informed by a broad spectrum of perspectives. This inclusivity extends to recognizing the interconnectedness of body, mind, and spirit and embraces a holistic view of human and environmental well-being.

As Aotearoa navigates its way through the complexities of the 21 st century, particularly in relation to Te Tiriti of Waitangi, the richness of its indigenous and settler cultures offers a formidable foundation for addressing global challenges. By using the Tiriti Relationship Framework to foster dialogue that is both respectful and forward-looking, we can lead the argument for the way diverse worldviews can shape the development of a shared vision for a sustainable and harmonious future. The pathway towards this future lies not in suppressing differences but in celebrating and weaving them into the fabric of national identity and purpose.

## Declarations

### Author declaration

All authors made substantial contributions to the conception and interpretation of this work, drafted and critically revised it for important intellectual content, approved the version to be published, and agree to be accountable for all aspects of the work, ensuring that any questions about its accuracy or integrity are properly addressed and resolved.
